# Locus coeruleus and the defensive activation theory of rapid eye movement sleep: A mechanistic perspective

**DOI:** 10.3389/fnins.2023.1094812

**Published:** 2023-02-23

**Authors:** Rasmus West Knopper, Brian Hansen

**Affiliations:** ^1^Center of Functionally Integrative Neuroscience, Department of Clinical Medicine, Aarhus University, Aarhus, Denmark; ^2^Sino-Danish Center for Education and Research, University of Chinese Academy of Sciences, Beijing, China

**Keywords:** locus coeruleus (LC), REM sleep, plasticity, neuroimaging, MRI

## Abstract

The defensive activation theory (DAT) was recently proposed to explain the biological function of dreaming. Briefly, DAT states that dreams are primarily visual to prevent plastic take-over of an otherwise inactive visual cortex during sleep. Evidence to support the DAT revolve around the interplay between dream activity (REM%) and cortical plasticity found in evolutionary history, primate studies, and coinciding decline in human cortical plasticity and REM% with age. As the DAT may prove difficult to test experimentally, we investigate whether further support for the DAT can be found in the literature. Plasticity and REM sleep are closely linked to functions of the Locus Coeruleus (LC). We therefore review existing knowledge about the LC covering LC stability with age, and the role of the LC in the plasticity of the visual cortex. Recent studies show the LC to be more stable than previously believed and therefore, the LC likely supports the REM% and plasticity in the same manner throughout life. Based on this finding, we review the effect of aging on REM% and visual cortex plasticity. Here, we find that recent, weighty studies are not in complete agreement with the data originally provided as support for DAT. Results from these studies, however, are not in themselves irreconcilable with the DAT. Our findings therefore do not disprove the DAT. Importantly, we show that the LC is involved in all mechanisms central to the DAT. The LC may therefore provide an experimental window to further explore and test the DAT.

## 1. Introduction

The question of why we sleep has puzzled mankind for centuries. For the brain, the function of sleep has long been thought to be mainly memory consolidation, but over the past decade or so, a clear physiological role has also been attributed to rapid eye movement (REM) sleep as a phase of increased clearance of waste products from the brain tissue (Xie et al., 2013; Lee et al., 2022). However, as of yet, no biological function has been attributed to another aspect of REM sleep, namely dream activity. It is, however, tempting to assign a biological function to dreams because of the brain’s famously tight energy budget (Attwell and Laughlin, 2001). If dream activity were a mere evolutionary remnant or a functionless byproduct of activity during REM sleep, it would seem too energetically costly to preserve. So why has evolution not done away with the night-time cinema as a cost-saving measure? To answer this, numerous attempts have been made to attribute functions to dreams. Aristotle was perhaps the first to take a neuroscientific view on dreams when he recognized dreams to “… follow the laws of the human spirit” (Beare, 1984) i.e., not being supernatural. Dreams have been seen as a window into our subconscious and have even been interpreted as a pre-language remnant of what Darwin termed our lowly origin (Darwin, 1871). In this view, dreams have been speculated to be the subconscious speaking in images merely out of ancient habit (McCarthy, 2017). Viewing the brain as a network, Crick and Mitchison (1983) instead proposed dreams to be a noise-based erasing mechanism termed reverse learning: “We dream in order to forget.” Broader, more mainstream theories state that dreams are the consequence of the brain actively sorting, storing, and processing input from the day’s activities (Kandel et al., 2021). Nevertheless, we still do not know why REM sleep produces dreams.

An intriguing idea presented by Eagleman and Vaughn (2021) proposes that dreams are primarily visual because REM sleep dreaming serves as a protective activation as otherwise long periods of sleep might cause an inactive visual cortex to be repurposed for other brain functions. Since our senses of touch, hearing, and smell are not shut off during sleep, the DAT would state that dreams need no or few auditory, olfactory, or tactile components as the cortical areas involved in these senses are not deprived of input during sleep. They coin their hypothesis the Defensive Activation Theory (DAT). As basis for their suggestion, Eagleman and Vaughn (2021) refer to studies demonstrating such a degree of cortical plasticity that input-deprived regions are taken over for other purposes after less than an hour of inactivity. This illustrates not only how plastic the cortex is but also that the brain uses this plasticity to provide on its surface a continually updated representation of the world based on the strength of sensory input to the cortex. One telling example of this phenomenon is the study by Merabet et al. (2007), where blindfolded volunteers practicing fine detail tactile discrimination showed touch-related activity in the visual cortex after about 45 min. Such recruitment of “unused” cortical areas has been widely demonstrated (Merabet and Pascual-Leone, 2010). Redistribution of the neural territory is not limited to the visual cortex but has also been demonstrated in the auditory cortex (Nishimura et al., 1999) and is often seen in patients as part of rehabilitation after stroke or sensory loss. Understanding this aspect of brain plasticity is, therefore, important not only for basic neuroscience but also in a clinical context. While certainly intriguing, the DAT may prove difficult to test experimentally. Instead, “directionally consistent” correlates are used to support the DAT. These examples draw on data from primates and evolutionary history but also studies of human brain. One specific example shows that a decline in human cortical plasticity with age happens alongside a decrease in dream activity with age. This agrees with the DAT because a less plastic cortex needs less defense from take-over. We will return to this central example throughout the present article, where we investigate whether existing data on relevant brain mechanisms provide other “directionally consistent” correlates to support or challenge the DAT. Specifically, our perspective aims to use existing literature on the human brain structure called the Locus Coeruleus (LC), REM%, and cortical plasticity to explore if these interconnected themes shed further light on the DAT. We also comment on neuroimaging methods that might be relevant for experimental study of the DAT.

## 2. The locus coeruleus and its relation to the DAT

The LC is a diffuse cluster of neurons symmetrically located on either side of the pons bordering on the fourth ventricle (Mouton et al., 1994; Ohm et al., 1997). In Man, the LC consists of 20,000–50,000 neurons. The LC produces most of the brain’s noradrenaline (NA). NA enhances the brain’s input response by heightening attention (Aston-Jones et al., 1991), and NA tone and concentration fluctuations aid memory formation and retrieval (Wagatsuma et al., 2018; Kjaerby et al., 2022). The LC projects to the entire brain and because NA is a neuromodulator, the LC exerts wide influence throughout the brain. It is known that LC provides trophic support to the brain through the NA network (Gesi et al., 2000) and is crucial for neuroplasticity (Glennon et al., 2019). To underscore this aspect, we note that optogenetic inhibition of LC has been shown to prevent stable memory formation (Wagatsuma et al., 2018). The LC is also involved in decision-making, sensory perception and processing, the physiology of correlated forebrain activity (ensembles and networks) (Sara, 2009; Poe et al., 2020), and the hemodynamic response (Giorgi et al., 2020).

Specifically, the LC is relevant to the DAT because of its involvement in the sleep-waking cycle (Aston-Jones and Bloom, 1981; Takahashi et al., 2010), REM-phase control (Hobson et al., 1975; Aston-Jones and Bloom, 1981; Khanday et al., 2016), and brain plasticity (Bear and Singer, 1986; Hu et al., 2007; Edeline et al., 2011; Martins and Froemke, 2015). All of these mechanisms are central to the DAT, or the arguments used by Eagleman and Vaughn (2021) to support the DAT. Therefore, it is natural to ask if the literature on the LC can add anything to the debate on the DAT. Following the example by Eagleman and Vaughn (2021), we survey the literature on LC to establish correlations and evaluate whether these are consistent with the DAT and the evidence provided in support of the DAT by Eagleman and Vaughn (2021). One such argument is the observation that REM sleep percentage (REM%, and thus dream activity) decreases with age in humans (Figure 4A; Eagleman and Vaughn, 2021) and that this is synchronous with a decrease in cortical plasticity with age (Figure 4B; Eagleman and Vaughn, 2021). This agrees with the DAT because a less plastic visual cortex is less likely to be taken over and, therefore, in less need of protective activation via dreaming. As described above, the LC is involved with both cortical plasticity (during non-REM sleep) and REM sleep onset which happens when REM-OFF neurons in the LC are silenced and NA concentrations decrease (Cespuglio et al., 1982; Kaur et al., 1997; Mallick et al., 2012). A logical question then is whether the LC is known to progress with age in a way that matches the age-dependent progression of REM% and cortical plasticity argued by Eagleman and Vaughn (2021).

### 2.1. Does LC decline with age? A survey of new evidence

Influential, early reports by German et al. (1988), Manaye et al. (1995) of either radiological data or post-mortem analysis showed a steady decline in total LC neuron count with age from a plateau of approximately 50,000 in the young adult to roughly 20,000 in the elderly. Most studies examining the age-related decline in LC neuron count suggest a reduction of 20–40% (Vijayashankar and Brody, 1979; Tomlinson et al., 1981; German et al., 1988; Lohr and Jeste, 1988; Marcyniuk et al., 1989) with selective cell loss of rostral cells compared to caudal cells (Chan-Palay and Asan, 1989; Manaye et al., 1995). Considering LC’s importance to brain physiology and cognition, age-related LC degeneration would have the consequence that brain deterioration with age is inevitable, likely also causing reduced cortical plasticity and decreased control of REM sleep consistent with the DAT.

However, some of these studies made lifespan comparisons on few brain samples with sample sizes ranging between 5 and 13 (German et al., 1988; Chan-Palay and Asan, 1989) and did not exclude cases with pathology elsewhere in the brain. More recent studies either excluding cases with neurofibrillary tangles (Mouton et al., 1994; Kubis et al., 2000) or using unbiased estimation procedures (Mouton et al., 1994; Ohm et al., 1997; Theofilas et al., 2017) found no age-related differences, suggesting that the healthy brain retains the LC during aging.

One reason for the confusion surrounding age-related LC size has to do with our current means of non-invasive LC size estimation. The locus coeruleus (from the Latin locus caeruleus, the blue spot) takes its name from the pigmentation of LC’s NA-producing neurons. This color comes from neuromelanin which is a byproduct of synthesis in catecholaminergic cell groups [cells that produce either of the neurotransmitters dopamine or NA (as with LC)] (Bogerts, 1981; Iversen et al., 1983; Baker et al., 1989). Neuromelanin chelates iron which may cause neuromelanin-rich cells to have MRI properties that stand out from the surrounding tissue (Sasaki et al., 2006; Clewett et al., 2016; Trujillo et al., 2017), making LC MRI dominantly sensitive to neuromelanin (Keren et al., 2015). Neuromelanin is known to accumulate over most of the human lifespan from early childhood until a plateau is reached at about sixty years of age. Consequently, neuromelanin-based MRI will have varying LC intensity over those six decades. NA has also been found to chelate iron (Singh et al., 2019), complicating interpretation of neuromelanin MRI further. Consequently, MRI for non-invasive assessment of LC is an immature technology because its signal-forming mechanism is not only unclear but also affected by multiple cellular mechanisms that are part of normal cell function, aging, and disease. This renders the data interpretation uncertain. Until better methods for non-invasive estimation of LC size arise, direct tissue analysis will continue to be the most reliable way to assess LC progression with age and in disease.

In summary, the newest literature points to the LC being a stable structure in the healthy brain. Changes in the LC, therefore, do not seem to underlie the age-dependent brain changes to cortical plasticity and REM sleep presented in support of DAT (Eagleman and Vaughn, 2021). Curiously, the LC exerts strong influence on the plasticity of the visual cortex (Bear and Singer, 1986), which is the territory defended by dreams according to the DAT. With the LC being stable with age and in control of visual cortex plasticity, it seems odd that the visual cortex would need less defense as we age. In the following, we briefly review the age dependence of cortical plasticity and REM for completeness.

## 3. Does REM sleep decline with age?

In the original paper, the DAT is supported with data showing correlations between REM% and expressions of neural plasticity (time to locomotion, weaning, and adolescence). As mentioned, the presented data show both REM% and cortical plasticity to decrease with age. Data from Roffwarg et al. (1966) was used to show a steady decline in REM% with age from roughly 30% at 19–30 years of age to approximately 22% in those aged 70–80 (Figure 4A; Eagleman and Vaughn, 2021). However, a more recent meta-study paints a somewhat different picture (Floyd et al., 2007). The study surveys 382 English-language research reports, thereby obtaining REM% values from 4,171 subjects. The overall finding shows that REM% is highly variable among subjects but the average is quite stable at 20–22% from the twenties to around 80 years of age. Only a slight linear decrease of 0.6% per decade was found up to the mid-70s, followed by a slight increase in REM% in the early 80s. Overall sleep duration decreases throughout life (Mander et al., 2017), and we, therefore, spend less time in both REM and non-REM sleep with age. However, REM% seems more robust to aging than what is presented in the original DAT paper. This does not challenge the DAT itself but does bring into question if the correlation between REM% and age used in support of the DAT agrees with the most recent evidence. This is relevant to point out because if the correlates shown by Eagleman and Vaughn (2021) are not representative of current knowledge, then new arguments in support of the DAT are needed for the theory to evolve.

## 4. The claim of decreased cortical plasticity with age

As mentioned, the LC has strong influence on brain plasticity. This influence is not simple as NA is known to influence the plasticity of different brain regions to different effects (Somarajan et al., 2016; Bari et al., 2020). For the DAT, the plasticity of the visual cortex is of main interest. Eagleman and Vaughn (2021) provide as support for the DAT the observation that cortical plasticity decreases with age, explaining why a decrease in REM% [shown by Roffwarg et al. (1966), discussed above] can be afforded without impairment of sight. The data shown in Figure 4B in Eagleman and Vaughn’s paper show a linear decrease in neuroplasticity in the motor cortex as measured by paired associative stimulation using EEG. This decline in plasticity is stated to be consistent with poorer recovery after damage in the aging brain. While no data of neuroplasticity in the visual cortex is shown in the DAT paper, the authors do briefly mention that repurposing of the visual cortex happens less with late loss-of-sight compared to those who are born blind, indicative that the visual cortex in the adult is less plastic than in the young. However, in the primary visual cortex, retention of plasticity is needed because visual recognition depends on it (Cooke et al., 2015). The review by Baroncelli and Lunghi (2021) similarly concludes that growing evidence shows that the adult visual cortex retains its neural plasticity more than previously thought and that the plastic potential of the visual cortex is preserved even with degraded visual input. This challenges the typical view of a critical period for visual cortex plasticity.

## 5. Discussion

Our survey focused first on the LC, which is known to be involved in the REM sleep, sleep-wake cycle, and brain plasticity. Eagleman and Vaughn (2021) present supporting data from evolutionary history, non-human primate brain and human brain. The LC is an ancient brain structure thought to have been involved in early amphibian diving response (Amaral and Sinnamon, 1977) and is quite similar in human and non-human primate brain (Manger and Eschenko, 2021). Nevertheless, we have limited the scope of our survey to the LC in the present-day human brain. The human LC was previously thought to decay with age but is now believed to be stable to old age in the healthy brain. Therefore, age-dependent changes to REM% and cortical plasticity do not seem to arise from the LC, which might have been assumed given the now abandoned view that LC decays with age. Next, we surveyed the literature to evaluate how a stable LC fits with the progression of cortical plasticity and REM% with age shown in support of the DAT. We find that the progression of both is more complex than presented in the original DAT paper. Specifically, we find that a recent meta-analysis including data from thousands of subjects shows REM% to be quite robust to aging. Furthermore, REM% increases in the early 80s. Finally, we found that the current literature suggests that the visual cortex retains its plasticity in old age to a much higher degree than previously thought. These findings are all consistent as a stable LC would be expected to sustain both REM% and visual cortex plasticity throughout life. Our survey confirms that the LC is involved in all mechanisms central to the DAT. If one were to speculate, our survey suggests that in the normal human brain REM% and visual cortex plasticity are stable over most of life, meaning that if the DAT is correct, then defensive dreams would be needed to continue in parallel. Our findings do not disprove the DAT but rather highlight where improved evidence for the DAT might be found. The understanding that the LC is involved in central mechanisms to the DAT may be important in the design of such experiments. We propose that the LC system may provide a way to test the DAT. If improved non-invasive means to assess LC integrity were developed, individuals with LC dysfunction could be sleep assessed and undergo measurements for visual cortex plasticity similar to the studies by Merabet et al. (2007). If LC dysfunction is followed by reduced visual cortex plasticity and decreased dream activity this would establish the LC as a potential biological seat for the DAT. We further note that neuroimaging methods exist that are capable of non-invasive detection of rapid cortical microstructure remodeling (Vukovic et al., 2021). With a better understanding of how the healthy brain ages, and the progression of sleep with age, such technology may be used to test the DAT further.

## 6. Conclusion

We argue that the LC is a central brain structure for the DAT with strong influence on mechanisms relevant to the DAT. Our survey shows that the canonical view of the LC as a structure undergoing decay with aging is likely not the true picture. Time-stability of the LC fits well with recent studies showing REM% to be quite stable across the lifespan and studies indicating that visual cortex plasticity is retained to a greater extent than previously known. While the findings of our survey contrast some of the human data originally provided in support of the DAT, they do not directly conflict with the DAT. As it stands, the DAT remains an intriguing idea about the possible biological function of the visual component of dreams.

## Data availability statement

The original contributions presented in this study are included in this article/supplementary material, further inquiries can be directed to the corresponding author.

## Author contributions

BH: conceptualization. RK and BH: writing. Both authors contributed to the article and approved the submitted version.
